# Analysis of the current transition readiness of adolescent patients with kidney disease and the factors influencing it

**DOI:** 10.3389/fped.2025.1613963

**Published:** 2025-09-19

**Authors:** Shuo Bai, Maomao Lv, Jinteng Liu, Wenjun Liu, Lili Jia

**Affiliations:** ^1^ Department of Pediatrics, Chinese People’s Liberation Army Eastern Theater Command General Hospital, Nanjing, China; ^2^ Nanjing Stomatological Hospital, Affiliated Hospital of Medical School, Institute of Stomatology, Nanjing University, Nanjing, China

**Keywords:** adolescents, kidney disease, transition readiness, current status, influencing factors

## Abstract

**Background:**

This study aimed to investigate the transition readiness of adolescent patients with kidney disease to understand their readiness status and ability to transition to adult healthcare and study the influencing factors, to provide a theoretical basis for improving the transition readiness of adolescent patients with kidney disease and developing intervention strategies.

**Methods:**

The study sample was obtained from the follow-up data of the pediatric department at the General Hospital of the Eastern Theater of Operations from December 2024 to January 2025, and patients aged 13–18 years with renal disease were selected as the study subjects. The scores of the Chinese version of the Self-Management and Transitional Readiness Questionnaire were used to determine the readiness status of the study subjects, and binary logistic regression was used to analyze the influencing factors.

**Results:**

A total of 242 questionnaires were distributed, and 12 invalid questionnaires were excluded, resulting in the inclusion of 230 samples for the analysis. Transitional readiness was at a low level in 123 cases (53.48%) and at a medium-high level in 107 cases (46.52%). The results of the binary logistic regression showed that the number of hospitalizations, treatment modalities, place of residence, caregiver's literacy, monthly income, and healthcare payment method were influencing factors for transition readiness in adolescent patients with kidney disease (*P* < 0.05).

**Conclusion:**

The readiness of adolescent patients with kidney disease during their transition to adulthood is not ideal and is affected by a combination of factors related to themselves, their families, and their socioeconomic status. The relevant medical personnel should implement a multidimensional approach and carry out targeted interventions to improve these patients’ readiness for the transition.

## Background

Chronic kidney disease (CKD) is a group of clinical syndromes characterized by structural damage and functional abnormalities of the kidneys, and its pathogenic mechanism involves multifactorial interactions (1). It is worth noting that epidemiological survey data show that the incidence of this disease in the pediatric population has shown a significant upward trend in recent years, and has become an important public health problem that threatens children's health (2). For adolescents with chronic kidney disease, the disease will accompany them throughout their growth, resulting in differences in their lives compared with the normal population. Clinical treatment of pediatric chronic kidney disease achieves partial remission, but there is a significant tendency toward relapse and risk of multisystem complications (3). Of particular concern is that long-term glucocorticoid regimens based on immunosuppression may trigger growth retardation, metabolic disorders, and other long-term endocrine system-related adverse effects, leading to an increasing tension between therapeutic benefits and developmental impairments (4). Studies of adolescents with chronic diseases have shown that the current healthcare system is characterized by intergenerational dependence, whereby patients rely primarily on their parents for disease monitoring and treatment decisions, and the lack of systematic transition preparedness education has resulted in patients failing to meet the desired standards of healthcare autonomy in the transition (5). This disruption in medical continuity significantly increases the risk of adverse clinical outcomes such as decreased treatment adherence, lack of self-management skills, and ultimately, accelerated disease progression and worsened long-term prognosis (6). One study showed that more than 50% of adolescents with chronic kidney disease were not prepared for transfer to adult care at the time of transfer (7). Currently, domestic research on the readiness of adolescents with kidney disease for the transition is still in its infancy, and there is a lack of research on the current readiness of the relevant population for the transition and the factors affecting it, with existing research only involving the exploration of the patient's inner feelings, behaviors, and perceptions (8). This study was a cross-sectional survey to understand the current readiness for transition among adolescents with kidney disease and the factors affecting it, with the aim of informing transitional care programs for adolescents with kidney disease.

## Methods

### Objectives of the study

Adolescent patients with nephropathy were selected for the study from the Department of Pediatrics, Eastern Theater General Hospital, from December 2024 to January 2025. The inclusion criteria were as follows: (1) meeting the diagnostic criteria for chronic kidney disease, (2) aged 13–18 years, and (3) informed parental consent to participate in this study. The exclusion criteria were as follows: (1) chronic kidney disease combined with severe cardiopulmonary and other organ failure, (2) chronic kidney disease combined with malignant tumors, (3) serious conditions that made it difficult to conduct investigations, and (4) hearing and speech communication disorders. All the patients’ disease information in this study was diagnosed and confirmed by physicians, and patient information was found in their medical records. The study was approved by the hospital’s ethics committee (2024DZKY-106-01).

### Investigation content relevant to this study

The Adolescent Kidney Disease Patient Profile Questionnaire provided data on the patients’ sex, age, education, type of disease, length of diagnosis, frequency of hospitalization, severity of disease, treatment, and symptoms, and whether they were an only child (Table 1). Furthermore, the Caregiver Profile Questionnaire for Adolescents with Kidney Disease provided data on the caregivers’ sex, age, place of residence, education level, occupation, relationship to the child, monthly family income, and mode of medical payment (Table 2).

**Table 1:** Distribution of the adolescents with kidney disease.

Variable	Cluster	Number	Percentage
Sex	Male	155	67.4
	Female	75	32.6
Age (years)	13–15	173	75.2
	16–18	57	24.8
Only child	Yes	71	30.9
	No	159	69.1
Education level	Junior high school	164	71.3
	Senior high school	66	28.7
Type of disease	Nephrotic syndrome	114	49.6
	Lupus nephritis	17	7.4
	Purpura fulminans	69	30.0
	Chronic kidney disease	30	13.0
Length of diagnosis (months)	<12	72	31.3
	13–24	35	15.2
	>24	123	53.5
Frequency of hospitalization	<5	178	77.4
	≥5	52	22.6
Severity of disease	Mild	193	83.9
	Moderate	28	12.2
	Severe	9	3.9
Type of treatment	Hormones	99	43.0
	Hormones + immunosuppressants	51	22.2
	Hormones + immunosuppressants + biologicals	18	7.8
	Other	62	27.0
Symptoms	Hematuria	28	12.2
	Proteinuria	100	43.5
	Hematuria + proteinuria	25	10.9
	Asymptomatic	77	33.5

**Table 2:** Distribution of caregivers of adolescents with kidney disease.

Variable	Cluster	Number	Percentage
Sex	Male	57	24.8
	Female	173	75.2
Age (years)	<45	181	78.7
	≥45	49	21.3
Place of residence	City	133	57.8
	Rural	97	42.2
Education level	Junior high school and below	97	42.2
	Senior high school	71	30.9
	College and above	62	27.0
Occupation	Labor	85	37.0
	Mental labor	80	34.8
	Unemployed	65	28.2
Relationship with the adolescent	Mother	167	72.6
	Father	53	23.0
	Other	10	4.2
Monthly household income (CNY)	<3,000	46	20.0
	3,000–5,000	76	33.0
	>5,000	108	47.0
Medical payment method	Medical insurance	127	55.2
	Self-financed	103	44.8

The Chinese version of the Self-Management and Transition Readiness Questionnaire Self-Management and Over-Preparedness Questionnaire (STARx) was designed by Ferris et al. (9) for adolescents with chronic illnesses to identify the main factors related to transition readiness. The Chinese version of the Self-Management and Transition Readiness Questionnaire (SMARTQ) was used in this study as a Chinese version of the STARx questionnaire, with 18 unchanged entries, and the original six dimensions were modified and integrated into the following four dimensions: medication management, health participation, doctor–patient communication, and knowledge of disease. The Cronbach's coefficient for the modified scale was 0.83. The questions in the questionnaire were scored on a 5-point Likert scale, with higher total scores indicating better transition readiness, and scores of less than 60 being defined as a low level of transition readiness in this study (10).

Finally, the Adolescent Kidney Disease Patient Profile Questionnaire and the Chinese version of the STARx were applied to the adolescent patients, while the Caregiver Profile Questionnaire for Adolescents with Kidney Disease was applied to the patients’ caregivers.

### Sample size estimation

**Figure 1:**
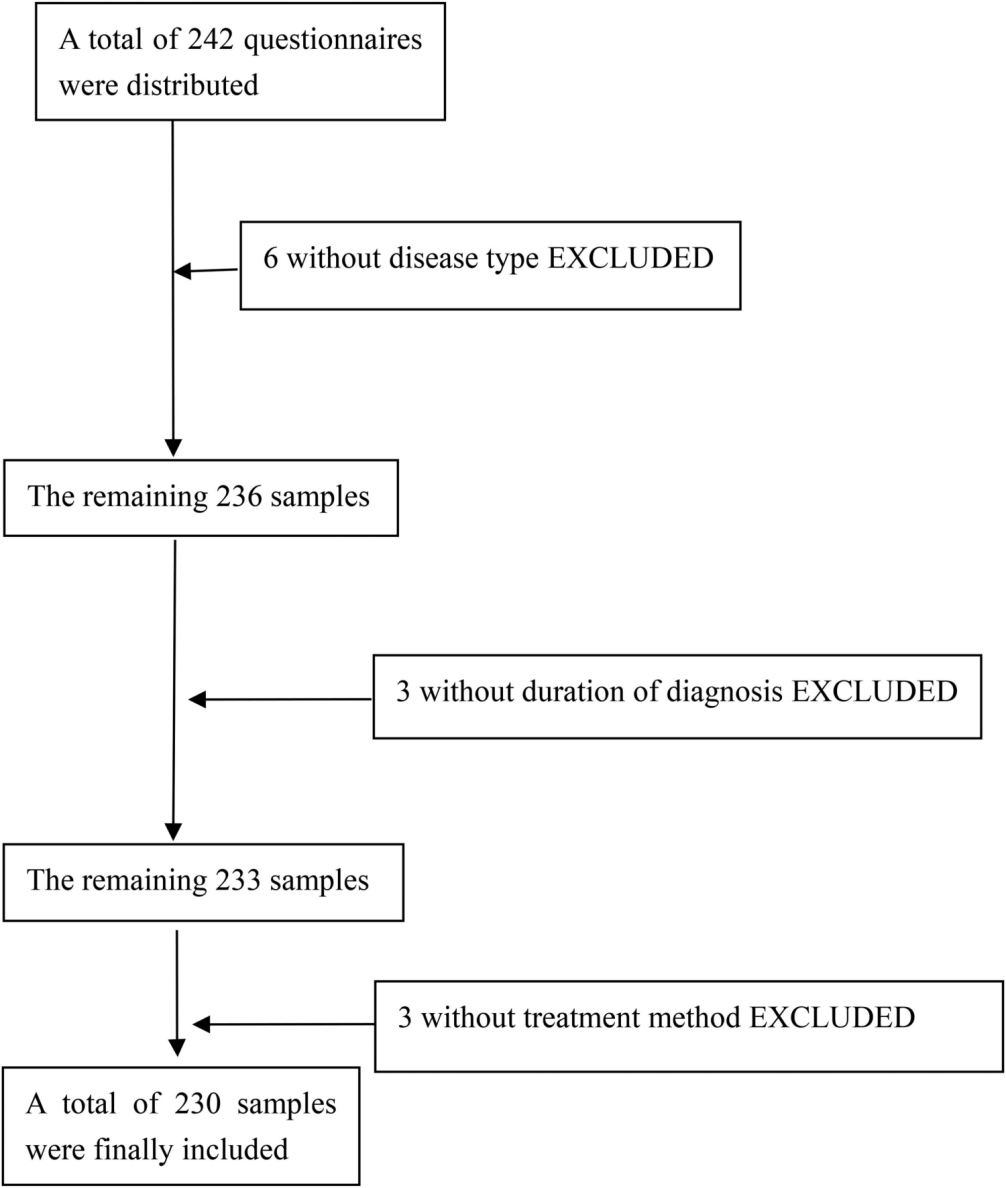
Flowchart of the data screening process.

Based on the relevant reviewed literature (11), a sample size of 5–10 times the total number of variables was required for this study. Thus, with a total of 22 variables included in this study, and taking into account about 20% of invalid questionnaires, a total of 242 questionnaires were distributed. Among the 12 excluded samples, 6 were lacking data in “disease type,” 3 were lacking data in “duration of diagnosis,” and 3 were lacking data in “treatment method” (Figure 1).

### Statistical methods

In this study, SPSS 27.0 software was used to statistically analyze the collected data. The count data were expressed as relative numbers and the χ^2^ test was used for the between-groups comparison. Measurement data with normal distribution were expressed as mean ± standard deviation and data with a non-normal distribution were expressed as M (P25, P75). Factors influencing transition readiness in adolescents with kidney disease were analyzed by binary logistic regression, and differences were considered statistically significant at *P* < 0.05.

## Results

### Univariate analysis of transition readiness in adolescents with kidney disease

Of the 230 adolescent patients with kidney disease, 123 (53.48%) had a low level of transition readiness and 107 (46.52%) had a medium-high level. Of those with a low level of transition readiness, 105 (85.36%) were in the 13–15-year-old age group, and 18 (14.64%) were in the 16–18-year-old age group. In the univariate analysis, the differences between adolescent patients with low and high levels of transition readiness were statistically significant (*P* < 0.05) for age, whether they were an only child, education level, length of disease diagnosis, number of hospitalizations, and mode of treatment; Moreover, for the caregivers, there were statistically significant differences in place of residence, education level, monthly income, and mode of medical payment (Table 3).

**Table 3:** Univariate analysis of transition readiness in adolescent patients with kidney disease.

Variable	Low level of preparedness (*n* = 123)	High level of preparedness (*n* = 107)	Χ^2^	*P*
Adolescents				
Age			14.61	<0.001
13–15	105	39		
16–18	18	68		
Sex			0.66	0.415
Male	80	75		
Female	43	32		
Only child			3.98	0.046
Yes	31	40		
No	92	67		
Education level			15.10	<0.001
Junior high school and below	101	63		
Senior high school	22	44		
Type of disease			7.60	0.055
Nephrotic syndrome	66	48		
Lupus nephritis	5	12		
Purpura fulminans	40	29		
Chronic kidney disease	12	18		
Length of diagnosis (months)			11.47	0.003
≤12	47	25		
13–24	23	12		
>24	53	70		
Frequency of hospitalization			21.90	<0.001
<5	110	68		
≥5	13	39		
Severity of disease			3.97	0.137
Mild	107	86		
Moderate	14	14		
Severe	2	7		
Type of treatment			25.72	<0.001
Hematuria	68	31		
Hormones + immunosuppressants	30	21		
Hormones + immunosuppressants + biologicals	6	12		
Other	19	43		
Symptoms			3.08	0.379
Hematuria	13	15		
Proteinuria	57	43		
Hematuria + proteinuria	16	9		
Asymptomatic	37	40		
Caregivers				
Age			0.15	0.697
≤45	98	83		
>45	25	24		
Sex			0.03	0.874
Male	31	26		
Female	92	81		
Place of residence			23.54	<0.001
City	53	80		
Rural	70	27		
Education level			17.50	<0.001
Junior high school and below	48	49		
Senior high school	39	32		
College and above	36	26		
Occupation			0.45	0.797
Labor	43	46		
Mental labor	44	32		
Unemployed	36	29		
Relationship with the adolescent			0.18	0.913
Mother	89	78		
Father	28	25		
Other	6	4		
Monthly household income (CNY)			29.38	<0.001
<3,000	41	5		
3,000–5,000	34	42		
>5,000	48	60		
Medical payment method			15.73	<0.001
Medical insurance	53	74		
Self-financed	70	33		

### Binary logistic regression analysis of transition readiness in adolescents with kidney disease

A binary logistic regression analysis was performed with the transition readiness of adolescent patients with kidney disease as the dependent variable and the statistically significant variables in the univariate analysis results as the independent variables (age, whether they were only child, education level, length of disease diagnosis, number of hospitalizations, and mode of treatment for the adolescent patients and place of residence, education level, monthly income, and mode of medical payment for the caregivers), which showed that the number of hospitalizations, treatment modalities, place of residence, caregiver's literacy, monthly income, and healthcare payment method were influencing factors for the transition readiness of adolescent patients with kidney disease (*P* < 0.05) (Tables 4, 5).

**Table 4:** Assignment of independent variables.

Variable	Assignment
Age	1 = 13–15; 2 = 16–18
Only child	1 = yes; 2 = no
Education level	1 = junior high school; 2 = senior high school
Length of diagnosis	1 ≤ 12 months; 2 = 12–24 months; 3 ≥ 24 months
Frequency of hospitalization	1 ≤ 5; 2 ≥ 5
Type of treatment	1 = hormones; 2 = hormones + immunosuppressants; 3 = hormones + immunosuppressants + biologicals; 4 = other
Place of residence	1 = city; 2 = rural
Education level of caregiver	1 = junior high school and below; 2 = senior high school; 3 = college and above
Monthly household income (CNY)	1 ≤ 3,000; 2 = 3,000–5,000; 3 ≥ 5,000
Medical payment method	1 = medical insurance; 2 = self-financed
Transition preparedness	1 = low; 2 = high

**Table 5:** Binary logistic regression analysis of transition readiness in adolescent patients with kidney disease.

Variable	B	SE	Wald χ^2^	*P*	Odds ratio (OR) (95% CI)
Age (years)					
13–15					1
16–18	−0.765	0.765	0.999	0.317	0.466 (0.104–2.084)
Only child					
Yes					1
No	0.009	0.416	0.000	0.984	1.009 (0.446–2.279)
Education level					
Junior high school					1
Senior high school	−0.969	0.705	1.891	0.169	0.380 (0.095–1.510)
Length of diagnosis (months)					
<12					1
13–24	0.247	0.459	0.290	0.590	1.280 (0.521–3.147)
>24	−0.236	0.552	0.182	0.670	0.790 (0.268–2.333)
Frequency of hospitalization					
<5					1
≥5	−2.013	0.565	12.701	<0.001	0.134 (0.044–0.404)
Type of treatment					
Hormones					1
Hormones + immunosuppressants	−5.122	1.249	16.810	<0.001	0.006 (0.001–0.069)
Hormones + immunosuppressants + biologicals	−2.556	1.139	5.035	0.025	0.078 (0.008–0.724)
Place of residence					
City					1
Rural	2.037	0.447	20.796	<0.001	7.666 (3.194–18.397)
Education level of caregiver					
Junior high school and below					1
Senior high school	1.112	0.517	4.621	0.032	3.309 (1.103–8.375)
College and above	0.122	0.497	0.060	0.806	1.130 (0.426–2.995)
Monthly household income (CNY)					
<3,000					1
3,000–5,000	−2.335	0.642	13.205	<0.001	0.097 (0.027–0.341)
>5,000	0.368	0.440	0.699	0.403	1.444 (0.610–3.419)
Medical payment method					
Medical insurance					1
Self-financed	1.494	0.398	14.091	<0.001	4.456 (2.042–9.722)

**Figure 2:**
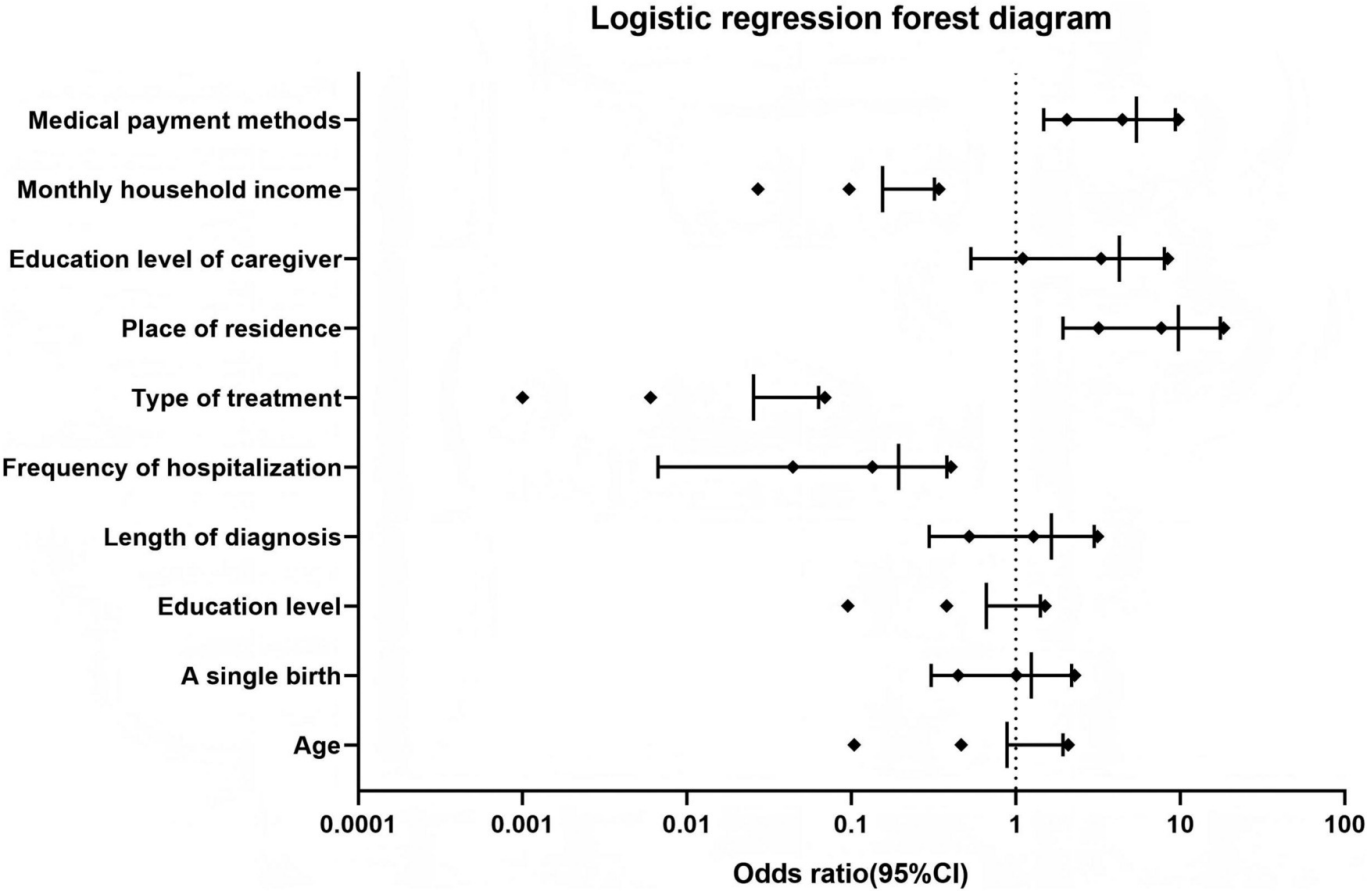
Forest diagram of the logistic regression analysis forest.

We assessed multicollinearity by calculating the variance inflation factor (VIF) for all the variables included in the multivariate logistic regression model. The VIF values for both age and education level were below the commonly accepted threshold of 1.0, indicating no serious multicollinearity. Finally, we created a forest plot summarizing the main results from the multivariable logistic regression analysis (Figure 2).

## Discussion

In this study, 53.48% of the adolescent patients with kidney disease had a low level of transition readiness, indicating that this problem has become a medical issue that cannot be ignored. This study adheres to the transitional guidelines of the European Society of Paediatric Nephrology (12).

In this study, adolescent kidney disease patients with ≥5 hospitalizations had lower transition readiness than those with <5 hospitalizations, a finding that differs from previous studies (13). Previous findings have suggested that patients with more hospitalizations have greater transition readiness because they have gradually adapted to their disease condition and have acquired some knowledge about their disease (14). The results of the present study are contrary to previous studies, and the reason for this phenomenon may be that frequent hospitalization of adolescent patients with kidney disease is caused by the complexity of the disease, poor control of the disease, or even deterioration of the disease, which results in adolescents having to rely on the decision-making of healthcare professionals for a long period of time, which weakens their own management (15). It may also be due to the fact that the medical care of the patient during hospitalization is led by healthcare professionals, and the patient is in the role of passive recipient of care for a long period of time, and lacks the opportunity to practice independent medication administration, symptom monitoring, etc., which leads to difficulties in adapting to being autonomous during the transition period (16). Therefore, healthcare professionals should pay attention to patients’ number of hospitalizations in daily patient management and develop their ability to participate in disease management from passive acceptance to active participation.

In this study, transition readiness was lower in adolescent patients with kidney disease treated with hormones + immunosuppressants or hormones + immunosuppressants + biologics compared to hormone therapy alone, which is consistent with previous findings (17). For patients, treatment complexity is positively correlated with cognitive load, and adolescent patients with kidney disease on multiple treatment modalities have to manage both hormonal treatment strategies and immunosuppressive blood level monitoring, with a management load that is significantly greater than that of patients on hormonal therapy alone (18). The risk of infection is greatly increased in patients treated with immunosuppressive drugs, and often the patient's daily social activities are compromised to avoid the risk of infection, leading to a decrease in social functioning and further dependence on parental decision-making (19). Co-medication can lead to an increase in the severity of a patient's disease, which can lead to negative self-perceptions and reduced motivation for self-health management in adolescent patients (20). This suggests that medical personnel involved in patient treatment and care should receive comprehensive education regarding the uses and precautions associated with medications. This will enable them to develop a thorough and clear understanding of their own medication regimens and disease conditions, thereby enhancing their confidence in treatment and helping to prevent negative or pessimistic attitudes.

In this study, the transition readiness of adolescent patients with kidney disease residing in rural areas was higher than that of patients in urban areas. It has been suggested that in rural areas, where there are more multigenerational families and a closer division of labor among relatives, patients may be involved in family affairs at an earlier age and develop independence and a sense of responsibility, which may enable them to take the initiative in disease management (21). Urban families may rely more on healthcare providers and have fewer opportunities for patient self-care, resulting in a lag in the development of self-management skills (22). Adolescent patients with kidney disease in rural areas are more adaptable to the independence requirements of the transition period because of the inconvenience of accessing medical care, which pushes them to learn medication management, disease monitoring, and some basic nursing care earlier; in urban areas, where healthcare resources are abundant, patients are accustomed to frequent visits and timely interventions, and there is a phenomenon of overprotection of children by their parents, which may neglect the development of long-term self-management skills (23). This leads to greater anxiety about independent living; therefore, transitional care should focus on fostering autonomy and designing differentiated support strategies for families from different backgrounds.

The results of this study showed that the transition readiness of adolescent patients with kidney disease with a monthly household income between $3,000 and $5,000 was lower than that of patients with a monthly household income of <$3,000, and that the transition readiness of patients who paid out-of-pocket was higher than that of patients who paid via medical insurance. Financial constraints force patients and families to acquire nursing skills at home earlier to reduce the financial burden of frequent visits to the doctor; thus, long-term financial pressure is an incentive for patients to take the initiative to learn disease management skills (24). Financially stressed patients and families may be forced to develop early recognition of symptoms and emergency management plans for their illnesses due to the inconvenience and cost of access, increasing their confidence to cope independently during the transition period (25). The experience of coping with resource scarcity over a long period of time leads to the development of problem-solving skills and resilience to stress, which are highly compatible with the psychological qualities needed for transition. Low-income patients, due to financial constraints, accept the reality of long-term coexistence with the disease earlier and incorporate self-management of the disease into their daily life planning (26). Higher-income families may become “service dependent” and rely more on paid services, resulting in less child autonomy and family engagement. Furthermore, higher-income families may be overprotective of their children, reducing their opportunities to face challenges and leading to weaker psychological adjustment. For patients covered by medical insurance, families may rely on insurance to a certain extent to cover costs, resulting in relatively low participation in the medical process. This may lead to patients lagging behind self-paying patients in terms of self-management skills and other areas, resulting in a slight lack of preparedness during the transition period. Self-pay means that families must bear the full cost of medical expenses, which makes them more attentive to the patient's condition and treatment process. Families may be more actively involved in treatment decisions, providing patients with greater support and guidance, thereby helping enhance patients’ understanding and management of their own conditions and prepare for the transition period. When managing adolescents during the transition period, healthcare professionals also need to be aware of the patient's family's financial situation and the child's caregiver's knowledge of the disease.

## Conclusion

This study shows that the overall level of transition readiness in adolescents with kidney disease is at a low level and is influenced by a combination of factors related to one’s self, family, and socioeconomic status. The results of this study suggest that transition readiness is not determined solely by resource abundance but is the result of a combination of resource access, stress coping patterns, and goal integration capabilities. The healthcare team needs to look beyond the surface and identify the mechanisms of family functioning to develop effective individualized transition plans, and future interventions need to focus on improving patients’ practical skills, psychological resilience, and community support networks.

## Data Availability

The original contributions presented in the study are included in the article/Supplementary Material, further inquiries can be directed to the corresponding author.
